# Glass half empty? Lessons learned about gastroparesis

**DOI:** 10.12688/f1000research.14043.1

**Published:** 2018-05-08

**Authors:** Patrick McKenzie, Klaus Bielefeldt

**Affiliations:** 1 Division of Gastroenterology, University of Utah, UT, USA; 2 Gastroenterology Section, George E. Wahlen Department of Veterans Affairs Medical Center, 500 Foothill Drive, Salt Lake City, UT 84103, USA

**Keywords:** Gastroparesis; macrophages; gastric emptying

## Abstract

Gastroparesis is defined as a combination of chronic dyspeptic symptoms and delayed emptying of a solid test meal. It remains a difficult-to-treat disorder with a significant impact on quality of life. Although gastroparesis is defined by delayed emptying, several important studies did not find a correlation between this biomarker and symptom severity or treatment success. Thus, some of the more recent trials explored strategies that ranged from antiemetics to antidepressants. Although dietary management showed benefit, most of the other interventions were barely superior to placebo or were not superior at all. Placebo responses were often quite high and this complicates the assessment of active agents. While it complicates the design and interpretation of clinical trials, high response rates for active and sham interventions indicate that we can achieve symptom relief in many patients and thus give them some reassurance. If indeed most therapies are only marginally better than placebo, the differences in adverse effects should be weighed more strongly, a point that is especially important in view of the controversy surrounding metoclopramide. Mechanistic studies introduced the network of macrophages as another potentially important player in the development of gastroparesis. Results are too preliminary and are largely based on preclinical data but show up- and downregulation of cellular elements controlling gastric function. Thus, future developments may teach us how they interfere with some of these mechanisms in clinical settings, potentially making gastroparesis a reversible process.

Eating not only ensures survival but also is part of the social fabric. Meals often function as times for social interaction, as symbols of hospitality, or as a central aspect of celebrations. Thus, illnesses that limit the ability to partake in this important daily activity have a disproportionately high impact on quality of life ^1^. About 60 years ago, Kassander coined the term gastroparesis, describing it as a consequence of vagal neuropathy in diabetics with secondary complications ^2^. We have since gained significant insight into the normal and abnormal function of the stomach, developed and standardized a disease definition and diagnostic approaches, and employed a wide range of treatments from diet to medications and endoscopic and surgical therapies. Current consensus statements highlight the importance of delayed gastric emptying, typically documented by measuring the exit of a solid test meal from the stomach, and the chronic presence of dyspeptic symptoms, such as nausea, vomiting, post-prandial fullness, early satiety, anorexia, and abdominal discomfort ^3^. Yet, given the repeatedly disappointing results of interventions that target disease-defining mechanisms, such as motilin or ghrelin agonists ^4– 8^, are we still facing a “conundrum”, as Longstreth lamented more than a decade ago ^9^? Since then, several investigations have shifted our understanding of the start of gastroparesis and this has affected clinical practice.

## Is it time to de-emphasize emptying time as endophenotype?

As mentioned above, gastroparesis is defined by a delay in gastric emptying, which differentiates it from functional dyspepsia or chronic unexplained nausea. The impaired emptying has traditionally been the target of treatments and often functioned as a key endpoint in clinical trials ^5, 6^. Yet gastric emptying does not consistently correlate with symptom severity, whether assessed with a labeled meal or a large undigestible particle, such as the wireless motility capsule ^10, 11^. This limited correlation could be due in part to a focus on the wrong endpoint, such as half-emptying time, rather than the fraction of a test meal retained after a predefined time ^12^. The relationship between gastric emptying and symptoms improved when acute, meal-induced rather than recall-based severity ratings covering a longer period were used ^13– 15^. These chronic complaints and acute, experimentally evoked symptoms are conceptually related; however, trial endpoints and drug approval criteria target the chronic symptoms as clinically validated and relevant endpoints. Interestingly, a recent cohort study described similar symptoms and symptom severity between type 1 and type 2 diabetics, even though gastric emptying was slower in type 1 diabetes, thus similarly pointing away from this variable as the sole or even primary determinant of disease severity ^16^. Conversely, the perceived benefit of treatments does not correlate with changes in gastric emptying ^17^. Lastly, gastric emptying does not reliably do what it is supposed to do as a biomarker, namely differentiate patients with overlapping symptoms but different disorders. Close to 90% of patients with gastroparesis meet the consensus definition of functional dyspepsia and about 20% of patients with functional dyspepsia have impaired emptying independent of their symptom pattern ^10, 18^. Similarly, a recent study characterized patients with chronic unexplained nausea, which phenotypically overlapped with gastroparesis except for the normal results of gastric-emptying studies ^19^. The overlap goes beyond symptoms and changes in emptying as detailed physiological studies showed coexisting changes in accommodation and sensory mechanisms in gastroparesis ^20^. If we look at mechanistic studies, we still see more parallels than differences as patients with otherwise-unexplained nausea and vomiting share some of the microscopic abnormalities on full-thickness gastric biopsies that otherwise have been associated with gastroparesis ^21^. Looking for alternative explanations related to gastric motor function, a small study suggested a potential correlation between symptoms and integrated motor activity ^22^, a finding that subsequent investigations did not confirm in a substantially larger cohort with 209 participants ^11^. Thus, we may deal with a spectrum of abnormalities that overlap in symptoms, test results, and underlying pathophysiology. Instead of making gastric emptying a key criterion for diagnosis and treatment, we may reconceptualize dyspeptic syndromes as a potential consequence of several coexisting mechanisms that contribute to a problem. Delayed emptying can clearly be one of these changes and may contribute to symptoms or symptom severity, especially the severity of nausea and vomiting ^18^ or post-prandial symptom exacerbations ^13– 15^. Some recent data also point to the potential prognostic value of gastric-emptying data with apparently counterintuitive results, as slower emptying at baseline was associated with a better prognosis after close to one year of follow-up ^23^.

## Will novel concepts bring novel treatments?

Animal models and human data have identified a network of macrophages in the muscular layer of the stomach, which may link the immune system to changes in gastric motor function. Activation of M1 macrophages triggers the release of tumor necrosis factor alpha, which, in turn, leads to a decrease in interstitial cells of Cajal (ICCs), the electrical pacemaker cells of the gut ^24^. Conversely, interleukin-10 activates M2 macrophages, which protect the ICC population ^25, 26^. With animal models showing up- or downregulation of ICCs through such mechanisms and associated changes in gastric emptying ^7^, do we need to look at this motility disorder as a problem at the interface between the immune system and neuromuscular transmission? Bharucha and colleagues recently explored this question by trying to induce heme oxygenase-1, which is expressed in M2 macrophages and plays an important anti-inflammatory role ^8^. Their pilot data show a potent induction of the enzyme but no influence on symptoms or emptying ^28^. Even if macrophages or specific enzymes have not yet found their way into successful treatment strategies, these findings illustrate how preclinical data are translated into novel treatment strategies. In addition, the underlying preclinical data may function like a ray of hope as they suggest that cellular abnormalities linked to the development of gastroparesis are potentially reversible.

One conceptual model envisions impaired opening of the pyloric channel as a cause for gastric retention, providing a rationale for treatments targeting this muscle. After two negative trials of botulinum toxin injection into the pylorus ^29, 30^, the potential role of pyloric dysfunction in gastroparesis has surfaced again in two forms. On the diagnostic side, the EndoFLIP (endolumenal functional lumen imaging probe) device allows detailed assessments of the pylorus’s active and passive mechanical properties, which seem to be altered in gastroparesis and appear to correlate with symptoms and emptying ^31, 32^. If this test is confirmed, we need to examine whether it enables us to identify a subgroup that could indeed benefit from interventions targeting this muscle structure. On the therapeutic side, per-oral endoscopic myotomy not only is feasible but also comes with impressive preliminary findings in early open-label trials ^33^. In light of the similar early enthusiasm with botulinum toxin therapy, followed by disappointing results in randomized controlled studies ^29, 30, 34– 37^, it is essential to integrate more detailed functional assessments of this muscle (for example, by using the EndoFLIP) and to assess the true impact of this intervention with controlled trials.

**Figure 1.**
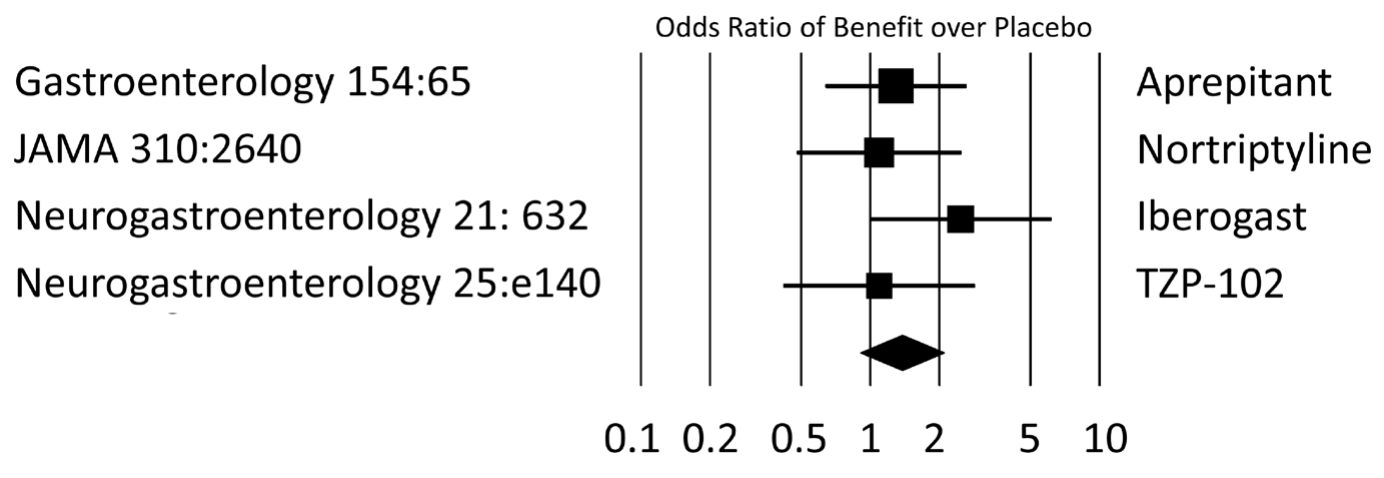
Summary of randomized controlled trials in gastroparesis with dichotomizing endpoints. Trials published within the last decade and providing response rates were assessed to determine the odds of active therapy being superior to placebo. All but the ghrelin agonist TZP-102 used symptom scores; TZP-102 compared gastric-emptying rates which were dichotomized based on half-emptying times of less or more than 150 minutes. Only the herbal preparation *Iberogast* was superior to placebo when the primary endpoint was analyzed. Studies are labeled on the basis of the publishing journal with volume and first page. JAMA, *Journal of the American Medical Association*; Neurogastroenterology, *Neurogastroenterology & Motility*.

**Figure 2.**
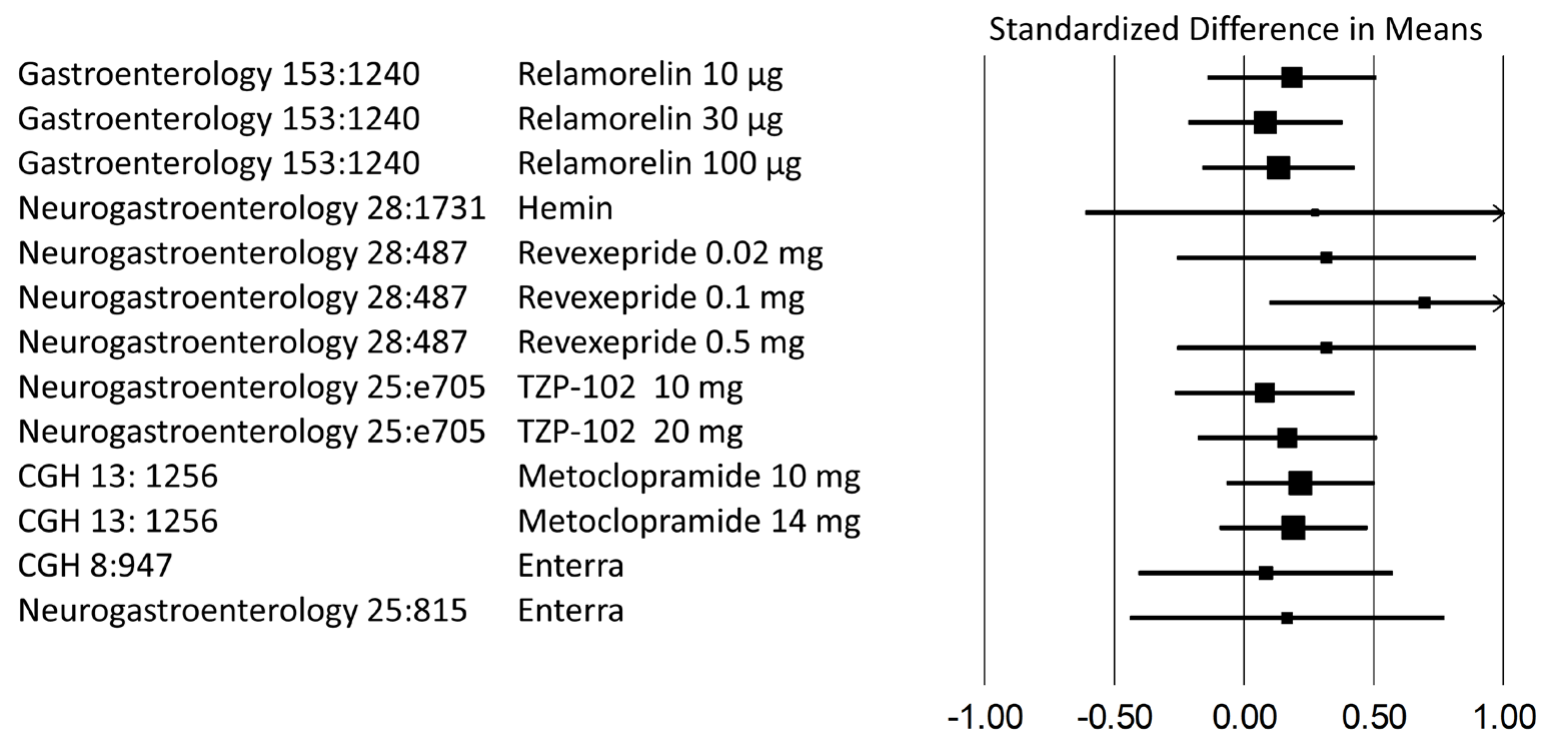
Differences in means between placebo and active therapy as shown in randomized controlled trials. Placebo-controlled trials published within the last decade were analyzed to depict differences in symptom scores between active interventions and placebo. All data are based on normalized mean changes in symptom scores. As the primary outcome variable for studies on gastric electrical stimulation (Enterra) was given only as median, we chose an aggregate score based on symptom frequency. CGH, *Clinical Gastroenterology and Hepatology*; Neurogastroenterology, *Neurogastroenterology & Motility*.

Several other observations about treatment options and impact have emerged during the last decade. First, a consortium of tertiary referral centers completed longitudinal studies that tracked symptoms and findings in patients who received treatment at the various sites for gastroparesis. Though not standardized and thus limiting our ability to assess specific treatment approaches, management was largely driven by the very experts who set the tone in discussions about the diagnosis and treatment of this illness. The data seem to be disappointing as even in expert hands, symptoms largely remain stable with less than one third of the patients improving during follow-up ^16, 23^. As already indicated, the observational design of this cohort study does not allow conclusions about specific steps. Yet it is important to contrast this message with the results of randomized controlled trials completed recently. We compiled data from double-blinded placebo-controlled trials published within the last decade and examined response rates for dichotomized endpoints ( Figure 1) or changes in severity ratings for continuous data ( Figure 2). Virtually all showed only marginal or even no differences compared with placebo. However, placebo responses were very high across the board, and vomiting frequency dropped by as much as 70%, to cite just one example ^38^. These results are difficult to reconcile with the largely stable symptoms reported by the consortium. In regard to one specific modality, gastric electrical stimulation, the benefit reported in retrospective case series and during the open-label trials, but not the blinded phase of controlled trials ^39– 41^, did not seem to translate into tangible changes for type 1 diabetics who often underwent device implantation as part of their management through members of the consortium ^16^. Most drug trials focused on ghrelin agonists, and short- but not long-term studies of one agent showed benefit over placebo ^7, 8, 42, 43^. A phase II trial of relamorelin, another ghrelin agonist, did not demonstrate improvement in vomiting as the primary endpoint when compared with placebo, but the active intervention was superior in an analysis of some secondary endpoints ^38^. After prior failures of motilin agonists ^4– 6^, another agent surfaced, again with promising results after a single dose in diabetics with gastroparesis ^44^. More appropriate long-term studies will be needed to see whether the most recent introduction is indeed different from the previously tested agents with a similar mechanism. For now, the most convincing standard therapy for gastroparesis is dietary intervention ^45^. Positive prognostic data, such as onset after an infectious prodrome, male gender, or antidepressant use, as well as negative prognostic factors, most notably opioid use and pain predominance ^23^, may enable providers to risk-stratify and tailor treatment approach or intensity. For providers outside of tertiary referral centers, the high placebo response rates and comparable effects with approaches based on very different mechanisms of action have important implications. If numbers needed to treat do not differ dramatically between therapies, the number needed to harm (that is, the side effect profile) becomes more important. While the effects of antiemetics and other agents commonly used in gastroparesis have been less systematically studied, the controversies surrounding metoclopramide clearly highlight the relevance of this criterion in gastroparesis ^46– 48^.

## Where to go from here

With at least some of the novel treatments not meeting expectations, gastroparesis remains a difficult-to-treat disorder. However, there have been clear advances in our understanding of this and perhaps some of the other closely related diseases. In our short review, we have tried to point to potential areas for future studies. One key question, is whether we should see gastroparesis as a distinct disorder or whether it belongs in the spectrum of syndromes labeled as functional dyspepsia, as has been suggested ^49^. Impaired motility clearly contributes to the pathophysiology of these common problems but includes more than just a delay in emptying. Thus, we need to assess whether other non-invasive tests, such as magnetic resonance imaging or single-photon emission tomography ^50, 51^, can provide a more comprehensive assessment of gastric motor function from accommodation to emptying and whether more detailed information allows more targeted and more effective therapy. If indeed emptying poorly correlates with symptoms and treatment effects, what can we offer beyond dietary advice? Recent studies give us some sense of direction, as anxiety and depression are associated with symptom severity ^10^ and as antidepressant use functioned as a positive outcome predictor ^23^. These results correspond to detailed mechanistic studies in functional dyspepsia that highlight the importance of affect ^52, 53^. Yet we still have to resolve some apparent contradictions, as well-designed trials led to inconsistent results with tricyclics not being superior to placebo in gastroparesis, while there was a benefit in a large cohort of patients with functional dyspepsia which included persons with impaired gastric emptying ^54, 55^. Going back to our call for a better and more comprehensive phenotypic characterization of patients, we may be able to identify subgroups with markers other than gastric emptying alone that may allow more targeted and more successful therapy. Concerns about metoclopramide led to a decreasing use of prokinetics with an apparent increase in antiemetics. Given the importance of nausea and vomiting as hallmark symptoms of gastroparesis, this development seems intuitively reasonable. However, the marginal benefit seen with aprepitant ^56^ demonstrates the need for evidence rather than intuition. Most of our insight into the efficacy of antiemetics comes from the treatment of chemotherapy-induced nausea, which is typically an acute and only transient problem. Although the clinical manifestation may look identical, the underlying mechanisms will almost certainly differ, forcing us to better define the true benefit of these agents in gastroparesis. Perhaps most importantly, we need to develop approaches that will enable us to provide better prognostic information and treatment for patients where they are most commonly seen, outside the tertiary referral centers.
